# Revolutions in treatment options in gastrointestinal stromal tumours (GISTs): the latest updates: Revolutions in treatment options in GIST

**DOI:** 10.1007/s11864-020-00754-8

**Published:** 2020-05-27

**Authors:** Sheima Farag, Myles J. Smith, Nicos Fotiadis, Anastasia Constantinidou, Robin L. Jones

**Affiliations:** 1 Sarcoma Unit, The Royal Marsden Hospital (NHS Foundation Trust) and Institute of Cancer Research, Fulham Road, London, SW3 6JJ UK; 2 Medical School University of Cyprus and BoC Oncology Centre, Nicosia, Cyprus

**Keywords:** Gastrointestinal stromal tumour, GIST, Tyrosine kinase inhibitors, TKI, Avapritinib, Ripretinib

## Abstract

The treatment of advanced GIST is rapidly evolving with the development of novel molecular compounds such as avapritinib and ripretinib, but also promising results have been achieved with cabozantinib in a phase II trial. The availability of over five lines of treatment for patients with advanced GIST is likely to completely shift the current second-line and third-line treatment options, and will also potentially enable a personalised approach to treatment. Imatinib will most likely remain as the first-line treatment of choice for the vast majority of GIST patients. However, for GIST patients with tumours harbouring a D842V mutation in *PDGFRA* exon 18, avapritinib has shown efficacy and will become first-line therapy for this molecular subgroup. For second- and third-line treatment, results are awaited of a number of clinical trials. However, second-line and further treatment could potentially be tailored depending on secondary mutations found in imatinib-resistant GISTs. As secondary resistance to TKIs remains the biggest challenge in the treatment of GIST and despite negative results with alternating regimens in phase II, combination treatments should be further evaluated to tackle this issue. Moreover, the favourable safety profiles observed with avapritinib and ripretinib suggest that combination treatments are feasible, for instance, combining two TKIs or a TKI with drugs targeting downstream signalling pathways, such as PI3K inhibitors or MEK inhibitors. Finally, in line with further personalisation of treatment in GIST, a multidisciplinary approach is essential, and local treatment options, such as RFA, resection in case of unifocal progression, and radiotherapy, should be considered.

## Introduction

Gastrointestinal stromal tumours (GISTs) are rare mesenchymal tumours that arise in the gastrointestinal tract, mainly in the stomach and small bowel. Around 90% of GISTs harbour driver mutations in *KIT* and platelet-derived growth factor alpha (*PDGFRA*) [1]. Since the introduction of imatinib, a tyrosine kinase inhibitor (TKI) that targets Brc-Abl (Philadelphia), KIT, and PDGFRA, the treatment of GIST patients with locally advanced and metastatic disease has dramatically improved. Median progression-free survival (PFS) in advanced disease is 20–24 months, overall survival (OS) is 5 years, and up to 85% of GIST patients with advanced disease have shown clinical benefit from imatinib [2].

The effect of TKIs depends on the type of mutation. For instance, in GISTs harbouring a mutation in KIT exon 9, imatinib 400 mg is less beneficial than imatinib 800 mg [3]. A D842V substitution in exon 18 of the *PDGFRA* gene shows primary resistance to imatinib at any dose [4]. Furthermore, GISTs that are wild type for *KIT* and *PDGFRA* are less sensitive and show primary resistance to imatinib treatment [5].

In case of progression or intolerance to imatinib, sunitinib is the recognised second-line treatment. Sunitinib is a multi-TKI that targets VEGFR, PDGFR, KIT, and CSF-1R. In a randomised phase III trial, of sunitinib 50 mg orally once daily for 4 weeks, followed by a 2-week period off drug in each 6-week cycle, the median PFS was 27 weeks compared with 6 weeks in the placebo arm [6]. Regorafenib, a TKI targeting VEGFR, KIT, PDGFR, FGFR, and RET, was registered in Europe in 2014 as an acknowledged third-line treatment in progressive disease. At a dosing schedule of 160 mg daily every 3 out of 4 weeks, regorafenib was demonstrated to improve PFS for up to 4 months in a phase III trial [7].

The role of local treatment options in metastatic GIST appears to be limited; however, retrospective studies have suggested that solitary progression in the context of otherwise controlled metastatic disease could be treated with radiofrequency ablation (RFA) or other modalities [8].

Resistance to treatment is the most significant challenge in the treatment of locally advanced and metastatic GISTs. Moreover, every TKI seems to have its own resistance pattern [9, 10]. Several studies are currently investigating different approaches to deal with this problem, and recently, novel molecular compounds have shown promising results in GIST patients whose disease is resistant to the currently available treatment options. In this manuscript, we provide an overview of the advances recently made in this rapidly evolving field.

## Avapritinib

Avapritinib is a drug that selectively targets activation loop mutations targeting *KIT* exon 17 and *PDGFRA* D842V mutations. This is important, since GIST patients harbouring these mutations, especially D842V in *PDGFRA* exon 18, show primary resistance to the currently approved TKIs.

The preliminary results of the phase I trial NAVIGATOR, presented at the Connective Tissue Oncology Meeting (CTOS) 2018, reported a total of 231 GIST patients with survival data being available for 207 patients treated in four different cohorts: (1) 20 GIST patients in second-line, (2) 23 GIST patients in third-/fourth-line regorafenib-naive, (3) 109 GIST patients in fourth or more advanced lines, and (4) 55 *PDGFRA* D842V-mutated GIST patients [11••]. Not all data were available at that time, hence the discrepancy in numbers. In the first 3 cohorts, the reported overall response rate (ORR) was 20–26%, with a median duration of response of around 7–10 months. In patients with GISTs with a *PDGFRA* D842V mutation, avapritinib resulted in more tumour shrinkage in almost all cases (98%), with an ORR of 84%. Besides, 9% of patients with a D842V mutation showed radiological complete response to avapritinib. These results are unprecedented in a disease known to be primarily resistant to imatinib and other approved TKIs. Data from the phase I trial resulted in the Food and Drug Administration (FDA) granting approval in January 2020 for this indication.

The phase III VOYAGER trial has recently completed the recruitment of 476 patients, investigating the efficacy of avapritinib compared with regorafenib in a 1:1 ratio in patients with advanced GIST progressing after 2 or 3 lines of treatment (ClinicalTrials.gov Identifier: NCT03465722). In April 2020, a press release regarding the Voyager trial reported no statistically significant difference in median PFS between the avapritinib arm (4.2 months) and the regorafenib arm (5.6 months). The overall response rate was 17.1% in the avapritinib arm and 7.2% in the regorafenib arm. The safety and tolerability of avaprinitib was similar to previous trials. Further analyses of these data are eagerly awaited, with particular attention to a potential subgroup of patients benefitting from avapritinib.

Safety data derived from the phase III trial revealed similar results as in phase 1 studies conducted with avapritinib. The most common toxicities with avapritinib are grade 1 or 2 and are in general manageable without the need to discontinue treatment. In the phase I study, most common grade 1 side effects were nausea (46%) and vomiting (30%), periorbital oedema (32%), increased lacrimation (28%), and hair colour changes (20%). Fatigue was the most common grade 2 toxicity (28%), with a total of 55% of patients experiencing fatigue of some degree. With regard to grade 3 toxicities, anaemia was reported in a quarter of patients [11••]. Unexpected neurocognitive side effects have been reported with the most common cognitive side effect being grade 1 memory impairment in 26% of patients, but also a decrease in mood has often been described. Neurocognitive side effects are managed with dose reductions or short interruptions and in most cases seem to be reversible [12].

## Ripretinib

This is a pan-KIT and PDGFRA switch control inhibitor. It has activity at both mutations occurring at the ATP-binding pocket and the activation loop. Prior in vivo and in vitro studies have shown promising results in GIST but also other cell lines (such as systemic mastocytosis, mast cell leukaemia, and germ cell tumours) transfected with *KIT* or *PDGFRA* mutants [13]. A phase I expansion study with GIST patients treated in (1) second line, (2) third line, and (3) fourth line and beyond is ongoing, but interim analysis of 114 patients revealed promising results. ORR was highest in patients treated in second line (24%), whilst in fourth line, the response rate was 9%. Disease control rate (DCR) was reported between 70 and 81% at 3 months with the lowest figure again in patients treated in the fourth-line setting [14].

A randomised phase III trial, INVICTUS, assessing efficacy and safety of ripretinib versus placebo in patients previously treated with imatinib, sunitinib, and regorafenib was presented at ESMO 2019. The trial met its primary endpoint after randomisation of 129 patients showing a statistically significant improvement in median PFS of 6.3 months for ripretinib compared with 1 month for placebo (HR 0.15, *p* < 0.0001) and median OS of 15.1 months (HR 0.36, *p* = 0.0004). It also confirmed the previously ORR of 9.4% shown in the phase I study cohort treated in fourth line and beyond [15••].

Additionally, ripretinib has demonstrated a largely favourable toxicity profile, with most toxicities being grade 1 or 2. Alopecia was the most common side effect in 51.8% of patients, followed by fatigue (42.4%), nausea (38.8%), abdominal pain (36.5%), constipation 34.1%), myalgia (31.8%), diarrhoea (28.2%), and decreased appetite (27.1%). Palmar-plantar erythrodysesthesia (PPE) syndrome was found in 21.2% of participants. The most common grade 3/4 adverse events were abdominal pain (7.1%), anaemia (9.4%), and hypertension (7.1%) [15••].

Considering its significant efficacy and tolerability in these patients with no approved treatment at present, ripretinib is expected to become the new standard of care for treatment in fourth-line therapy and beyond. Currently a phase III study, INTRIGUE, is recruiting patients to assess the efficacy and safety of ripretinib compared with sunitinib in second line after progressing on imatinib [16].

## Cabozantinib

Cabozantinib is a novel compound that targets MET, VEGFR2/KDR, RET, KIT, AXL, and FLT. MET inhibition caused by cabozantinib is believed to possibly overcome upregulation of MET signalling as a result of imatinib inhibition of the KIT pathway [17]. The drug is proven active in patient-derived GIST xenografts and was associated with patient benefit in imatinib- and sunitinib-resistant GIST patients in a Japanese phase I trial including 4 GIST patients [18, 19]. In preclinical studies, cabozantinib has proven to be effective in both imatinib-sensitive xenografts and imatinib-resistant models [19].

The CaboGIST trial is a phase II study investigating the efficacy of cabozantinib in patients progressing after treatment with imatinib and sunitinib. It is now closed after inclusion of 50 patients and met its endpoint with 58.8% of patients being progression free at 12 weeks [20•]. Median PFS was 5.5 months (95% CI 3.6–6.0), and OS was 18.2 months (95% CI 14.3–22.3). Objective response (OR) was found in 7 out of 50 patients (14%), and clinical benefit, including those patients with stable disease, was observed in 41 patients (82%). Interestingly, Objective responses were noted in tumours harbouring both KIT exon 11 and 17 mutations. Clinical benefit (PR or SD as best response) was found in a wide range of mutations, including *KIT* exon 9, exon 13, and exon 14. Also, GIST patients with neurofibromatosis type 1 (NF1) were entered in this trial and had stable disease as best response [20•].

The safety profile of cabozantinib was considered acceptable; however, the most common grade 3 side effects were diarrhoea and hypertension, reported in 26% (*n* = 13) and 36% (*n* = 18), respectively. PPE was observed in 60% of patients and was mostly grade 2 or less. Fatigue grade 1/2 was found in 50% of patients. Other common grade 1/2 side effects were mucositis (30%), anorexia (24%), abdominal pain (22%), hypothyroidism (20%), hoarseness of voice (18%), dysgeusia (18%), skin rash (16%), nausea (12%), and myalgia (12%). These results found in a wide variety of mutated GISTs are very promising and warrant further investigation of cabozantinib in advanced GIST.

## Combination treatments

Secondary resistance as a result of exposure to TKIs is a main challenge in the treatment of GIST. There is increasing evidence that every TKI has its own resistance profile. One potential strategy to manage this problem is to alternate between two different TKIs with accompanying variations in targets. ALT-GIST is a phase II randomised non-comparative trial to determine if an alternating regimen of imatinib and regorafenib has sufficient activity and safety to warrant further evaluation as a first-line treatment for metastatic GIST. Patients were treated with 21–25 days of imatinib 400 mg followed by a 3- to 7-day gap and started again on regorafenib 160 mg for 21 days with a 7-day washout period before starting the next cycle with imatinib. The control arm was provided with imatinib 400 mg continuously. The primary endpoint was objective tumour response (OTR) at 9 months [21•]. Interim analyses in 76 patients revealed no meaningful difference in the primary endpoint between the two groups with OTR being 60% (95% CI 45–74%) in the intervention arm and 64% (95% CI 48–78%) in the control arm. Furthermore, no unexpected intolerance was found. The study is ongoing, and other endpoints will be reported in due course [21•].

Another trial investigating the safety and efficacy of alternating treatment regimens in GIST is a phase I study rapidly alternating sunitinib with regorafenib. In this study, 14 TKI refractory patients were given sunitinib for 3 days followed by regorafenib for 4 days. Patients entered the study have been treated in third line and beyond. In these patients, the regimen was deemed tolerable, and no unexpected toxicity occurred. However, no objective response was found, and 4 out of 13 evaluable patients have shown stable disease as best response [22•]. Despite the less convincing antitumour responses documented, the study shows that rapid alternation of different TKIs is feasible and safe, leading to the potential evaluation of a similar paradigm using other TKIs proven to be more effective in resistant GIST patients.

Besides alternating treatment regimens, combination treatments have been assessed using phosphoinositide 3-kinase (PI3K) inhibitors such as BKM120 or BYL719. In preclinical animal studies, imatinib combined with PI3K inhibitors has shown better responses than treatment with imatinib as single agent. Volume reduction was shown to be more dramatic, and apoptosis was enhanced in the mice receiving combination treatment [23]. Following these results, a number of phase I studies have been initiated, assessing the safety and efficacy of PI3-kinase inhibitors. Results are currently awaited. A recent phase I study investigating the PI3K inhibitor buparlisib with imatinib in patients who progressed following treatment with imatinib and sunitinib. The study did not show convincing responses to treatment, however this does warrant further research in combination treatment regimens [24].

Furthermore, in a study conducted to assess resistance mechanisms in GIST, 18% have shown a *KIT* downstream mutation [10]. The same study suggested that combination treatment with mTOR or MEK inhibitors might be required to overcome the non-KIT-related resistance mechanisms in GIST. Currently, several phase I/II trials are investigating the efficacy of combination of MEK inhibitors in treatment of GIST [16]. In other tumour types, such as (mucosal) melanoma, this paradigm has proven to be effective [25].

## Imatinib reintroduction

In a phase III randomised placebo-controlled (RIGHT) trial investigating the response to imatinib in GIST patients who progressed through imatinib and sunitinib, imatinib rechallenge has shown little benefit with median PFS of 1.8 months in the imatinib arm compared with 0.9 months in the placebo arm (HR 0.46, 95% CI 0.27–0.87, *p* = 0.005) and OS of 8.2 months versus 7.5 months, respectively [26•]. Of the 81 patients included in the study, over 90% progressed within 4 months, and no ORR was observed [27]. Disease control rate (DCR) at 12 weeks however was 32% in the treatment arm versus 5% in the placebo arm (*p* = 0.003) [26•].

## A personalised approach for the management of GIST

Despite the promising results that have been observed with novel treatments, such as avapritinib and ripretinib, imatinib remains the first-line treatment of choice for a large majority of GIST patients. Due to the efficacy of avapritinib in advanced *PDGFRA* exon 18 D842V-mutated GIST, this agent should be offered as first-line therapy to those harbouring this mutation. For the second-line treatment, the results of the phase III INTRIGUE trial, comparing ripretinib versus sunitinib, are awaited. For third-line treatment, top-line results of the phase III VOYAGER trial comparing avapritinib to regorafenib has shown no statistical difference in PFS, although ORR was higher in the avapritinib cohort. A recent review has suggested a complete shift in second-line and third-line treatment with sunitinib and regorafenib being replaced by ripretinib and avapritinib [28]. In the same publication, an alternative flow chart is presented in which both drugs are followed by ‘other tyrosine kinase inhibitors’ in fourth line and beyond [28]. However, the overwhelming responses of PDGFRA D842V-mutated GISTs to avapritinib were not included. Besides, we believe that the potential availability of over 5 lines of treatment for advanced GIST warrants a more personalised approach in keeping with the most recent advances made in drug development and insights gained in resistance mechanisms.

**Fig. 1:**
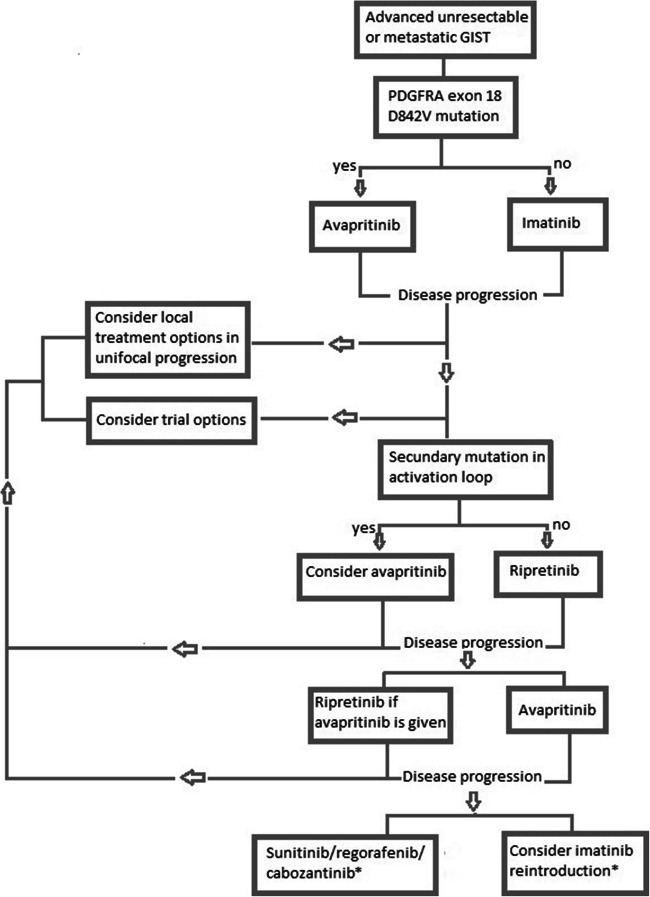
Suggested potential treatment paradigm based on personalised approach following the latest advances in the field. *Beyond third-line patients’ performance status and comorbidities should be considered.

In Fig. 1, we present a flow chart in which a tailored treatment paradigm is proposed based on the primary and secondary mutations found in patients showing progressive disease. Sequencing of tumour material or liquid biopsies assessing circulating tumour DNA (ctDNA) might be used to assess the specific resistance mechanisms in the individual patient to find potentially targetable mutations preferably in a trial [10, 22•, 29–31]. Despite the negative results of the phase III VOYAGER trial, avapritinib can still be considered as third-line and even second-line treatment option. Secondary mutations, symptomatic progression and patients' perfomance score should be considered as ORR was higher in the avapritinib treated cohort and the drug has shown to be well tolerable. Trials with combination treatments are expected to inhibit secondary resistance mechanisms in patients treated in second-line and third-line treatment and should be given priority in the near future [16].

In patients with unifocal progression, several studies in a preselected cohort have shown that surgical removal or ablative therapy of the progressive site leads to improvement in PFS [32, 33]. Data on the efficacy of surgery in metastatic GIST are limited, and no prospective studies have been conducted. In a recent publication, it has been suggested that oligometastatic GIST patients responsive to systemic treatment may be considered for resection with a possible improvement of PFS [34]. In particular in patients for whom further lines of treatment are available, surgery should be furthermore considered for symptomatic or complicated cases, such as GISTs causing fistulation or obstruction. Surgery should hence be discussed within the context of a multidisciplinary team. In patients with bone metastases, radiotherapy might be beneficial [32]. For many years, radiotherapy was considered not to have a prominent role in the treatment of GIST. However, several case series suggest that radiation may have a role in advanced GIST [35, 36].

Several studies have investigated the role of therapeutic drug monitoring to assess inter-patient and intra-patient variability in exposure to TKIs [37, 38]. One study has shown a correlation between response to imatinib and imatinib exposure [39]. Also, previous studies have found a dose-effect relationship in GIST patients treated with sunitinib [40]. These studies indicate that TDM might improve efficacy and tolerance of TKIs in GIST.

Finally, a personalised approach should incorporate evaluation of the performance status and past medical history of an individual patient. Despite the relatively small benefit found in patients treated with imatinib rechallenge after initial progression, patients with a poor performance status or elderly patients might benefit from imatinib rechallenge in combination with best supportive care. It might not completely halt the disease, but it is believed to at least slow disease progression and may improve symptoms [26•].

In conclusion, recent advances made in the treatment of GIST are very promising and warrant further trials and personalised treatment within the context of a multidisciplinary approach. Specifically, ripretinib and avapritinib have shown very promising results and may change the current second- and third-line treatment paradigm and beyond. Moreover, in TKI-resistant GISTs, such as those harbouring a *PDGFRA* exon 18 D842V mutation, a promising first-line treatment option is now available.
